# First-line treatment options for PD-L1-negative lung adenocarcinoma: a real-world analysis

**DOI:** 10.3389/fonc.2025.1533048

**Published:** 2025-06-04

**Authors:** Lijuan Chen, Jie Liu, Junfeng Lu, Xiufeng Hu, Erjing An, Yanqiu Zhao

**Affiliations:** ^1^ Department of Oncology, The Affiliated Cancer Hospital of Zhengzhou University & Henan Cancer Hospital, Zhengzhou, China; ^2^ Henan International Joint Laboratory of Lung Cancer Biology and Therapeutics, Zhengzhou, China

**Keywords:** lung adenocarcinoma, PD-L1 negative, first-line treatment, bevacizumab, immunotherapy, prognosis

## Abstract

**Objective:**

To evaluate the optimal first-line treatment options for programmed death-ligand 1 (PD-L1) negative lung adenocarcinoma (LUAD) patients without common gene-alterations.

**Methods:**

A total of 159 PD-L1-negative LUAD patients without common gene-alterations were included. Chemotherapy was administered in 44 cases (group A), immunotherapy-chemotherapy combinations in 55 cases (group B) and bevacizumab plus chemotherapy in 60 patients (group C). A head-to-head comparison of the clinical effectiveness and safety for these standard treatment regimens was conducted.

**Results:**

The median follow-up time was 30.9 months. For the entire cohort, median PFS was 6.67 months [95% CI 5.83-7.51], and median OS was 15.83 months [95% CI 13.46-18.21]. OS was significantly longer in group C versus others (C vs B median 21.6 months [95% CI 17.78-25.42] vs 12.63 months [95% CI 8.14-17.13]; HR 0.59 [95% CI 0.39-0.9], P = 0.01; C vs A median 21.6 months [95% CI 17.78-25.42] vs 13.47 months [95% CI 9.68-17.26], HR 0.47 [95% CI 0.3-0.71], P= 0.001), but no substantial difference was noted between group A and B (HR 0.78 [95% CI 0.51-1.2], P= 0.26). For PFS in pairwise comparison, group B and C were statistically superior to group A (B vs A median 5.6 months [95% CI 4.56-6.64] vs 5.17 months [95% CI 4.09-6.25]; hazard ratio (HR) 0.56 [95% CI 0.37-0.87], P = 0.009; C vs A median 8.57 months [95% CI 7.47-9.66] vs 5.17 months [95% CI 4.09-6.25]; HR 0.43 [95% CI 0.28-0.7], P< 0.001), whereas no significant difference was found between group B and C (HR 0.76 [95% CI 0.51-1.11], P= 0.16). The disease control rate (DCR) improvement was sustained with group C (A vs B vs C: 84.09% vs 83.64% vs 96.67%, respectively (P<0.05)). Multivariate analysis showed that the performance status score and treatment regimen were factors influencing PFS as well as OS. The treatment-emergent adverse events (AEs) of grade 3–4 occurred in a similar proportion of patients in each group (P>0.05), and all AEs were manageable without fatal toxicities.

**Conclusions:**

Bevacizumab plus chemotherapy should be prioritized in PD-L1-negative LUAD patients without common driver gene alterations. These findings may facilitate individualized treatment options.

## 1 Introduction

Lung cancer is a leading cause of cancer incidence and death, with lung adenocarcinoma (LUAD) being its most dominant histological subtype (1, 2). As most LUAD are diagnosed at an advanced stage, disease management can be challenging (2). Prior to the availability of targeted therapies, median survival for patients with advanced LUAD was only seven to eight months, despite aggressive platinum-based chemotherapy (3). Bevacizumab was among the first targeted therapies available for this cancer and the first recombinant humanized monoclonal antibody against vascular endothelial growth factor (VEGF) to help the patients live longer than one year when added to chemotherapy. Approval in the first-line setting was based on the results of the pivotal study ECOG4599 which demonstrated a reduction in the risk of death by 21% (HR: 079, p=0.003) with the addition of bevacizumab to chemotherapy compared chemotherapy alone and improvement in median overall survival (OS) from 10.3 to 12.3 months (4). Further clinical trials confirmed the survival benefits for bevacizumab in combination with chemotherapy in the first-line treatment of LUAD (5, 6).

In recent years, immune-checkpoint inhibitors (ICIs), specifically those targeting programmed cell death protein 1 (PD-1) or programmed death-ligand 1 (PD-L1), have led to a paradigm shift in the first-line treatment landscape of LUAD, making long-term survival possible (7). Immunotherapy regimens based on ICI therapy, combined with platinum-based chemotherapy have become the standard of care as a first-line treatment for advanced LUAD without sensitizing EGFR or ALK mutations (8, 9). However, only 20-40% of patients derive benefit from these new therapies (8). Indeed, the extent of tumor cell PD-L1 expression is paramount to ICI selection, thus ICI monotherapy or ICI plus chemotherapy are more recommended for those patients with PD-L1 positive expression (7–11). Nevertheless, for patients who were negative for tumoral PD-L1 expression, the efficacy of immunotherapy as the first-line treatment is unclear. Most importantly, this group accounts for about half of the whole patient population (12).

For advanced LUAD patients who present with no known driver mutation and are negative for tumoral PD-L1 expression, first-line treatment strategies include chemotherapy, chemotherapy plus bevacizumab, and chemotherapy-immunotherapy combinations. Many studies demonstrated that chemotherapy combined with ICIs or bevacizumab resulted in better outcomes compared to the use of chemotherapy alone in treatment-naive advanced LUAC patients without known driver mutations (4–13). However, head-to-head comparisons of these treatment regimens in PD-L1-negative patients remain limited. In this study, clinical data from first-line treatment in PD-L1-negative LUAD patients without common driver gene alterations was retrospectively analyzed. The efficacy and safety of platinum-based chemotherapy, with addition of immunotherapy or bevacizumab were evaluated in head-to-head comparison, and the clinical characteristics of the beneficiaries of different treatment plans were analyzed. The present study provides a basis for selecting the optimal first-line therapeutic management for PD-L1-negative LUAD in patients without common driver gene alterations.

## 2 Materials and methods

### 2.1 Study design

**Figure 1:**
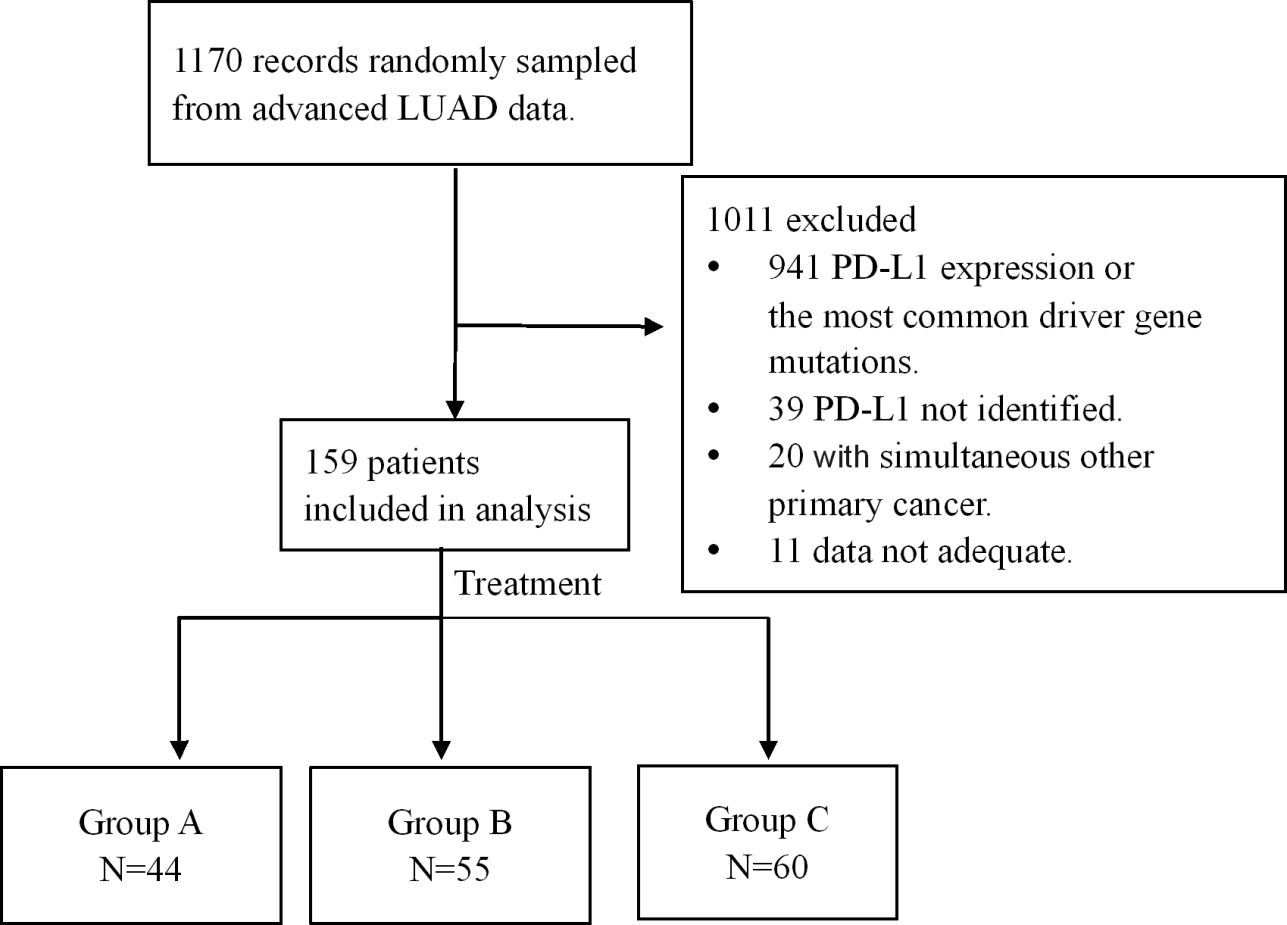
Selection process and enrollment. 1170 medical records of advanced LUAD were randomly sampled from the database, and 159 patients were enrolled. Among the 159 patients, chemotherapy was administered in 44 cases (group A), immune-checkpoint inhibitors (ICIs) in combination with chemotherapy in 55 cases (group B) and bevacizumab plus chemotherapy in 60 patients (group C).

1170 medical records of advanced LUAD were randomly sampled from the database of Henan Cancer Hospital from January 2015 to May 2023. Eligibility criteria for inclusion in this study were as follows: (1) Confirmed to have advanced LUAD by histopathology and treated with platinum-based chemotherapy or platinum-based chemotherapy combined with ICIs or bevacizumab as the first-line treatment plan; (2) Age > 18 years old, regardless of sex; (3) Eastern Cooperative Oncology Group performance status (ECOG PS) score of 0–2 points; (4) Received at least 2 cycles of first-line regimen treatment, and the efficacy was evaluated; (5) Without sensitizing EGFR, ALK, ROS1, Met, Ret or Braf V600E mutations. Exclusion criteria: (1) Small cell lung cancer, squamous cell carcinoma, or undifferentiated type; (2) Multiple primary tumors. The selection process is shown in **Figure 1**.

A total of 159 patients with advanced LUAD lacking common driver gene alterations and tumoral PD-L1 expression were retrospectively analyzed. All patients provided informed consent, and the study was approved by the Ethics Committee of Henan Cancer Hospital. Treatment regimens included chemotherapy alone (Group A, n = 44), chemoimmunotherapy (Group B, n = 55) or bevacizumab plus chemotherapy (Group C, n = 60). Patient disposition is detailed in **Figure 1**, and baseline characteristics are summarized in **Table 1**.

**Table 1:** Baseline demographic and clinical characteristics.

Characteristics	Total	Group A	Group B	Group C	χ²	P
	(N=159)	(N=44)	(N=55)	(N=60)		
Age, years	61 (32-85)	61 (32-81)	64 (44-81)	59 (36-81)	3.91	0.14
<65	100 (62.89%)	29 (65.91%)	29 (52.73%)	42 (70.00%)		
≥65	59 (37.11%)	15 (34.09%)	26 (47.27%)	18 (30.00%)		
Sex, n (%)					2.54	0.28
Male	112 (70.44%)	30 (68.18%)	43 (78.18%)	39 (65.00%)		
Female	47 (29.56%)	14 (31.82%)	12 (21.82%)	21 (35.00%)		
Tabacco use history, n (%)					3.99	0.14
Current or former smoker	89 (55.98%)	24 (54.55%)	35 (63.64%)	32 (53.33%)		
Never smoked	70 (44.02%)	20 (45.45%)	20 (36.36%)	28 (46.67%)		
ECOG, PS n (%)					3.28	0.19
0-1	136 (85.53%)	38 (86.36%)	44 (80.00%)	55 (91.67%)		
≥2	23 (14.47%)	6 (13.64%)	11 (20.00%)	5 (8.33%)		
ERBB2 mutation, n (%)					0.43	0.81
Positive	13 (8.18%)	3 (6.82%)	4 (7.27%)	6 (10.00%)		
Negative	146 (91.82%)	41 (93.18%)	51 (92.73%)	54 (90.00%)		
Kras mutation, n (%)						
Positive	27 (16.98%)	6 (13.64%)	13 (23.64%)	8 (13.33%)	2.64	0.27
Negative	132 (83.02%)	38 (86.36%)	42 (76.36%)	52 (86.67%)		
TP53 mutation, n (%)						
Positive	27 (16.98%)	4 (9.09%)	12 (21.82%)	11 (18.33%)	2.93	0.23
Negative	132 (83.02%)	40 (90.91%)	43 (78.18%)	49 (81.67%)		
Liver metastasis						
Yes	24 (15.09%)	7 (15.91%)	10 (18.18%)	7 (11.67%)	0.98	0.61
No	135 (84.91%)	37 (84.09%)	45 (81.82%)	53 (88.33%)		
Bone metastasis						
Yes	56 (35.22%)	21 (47.73%)	20 (36.36%)	15 (25.00%)	5.8	0.06
No	103 (64.78%)	23 (52.27%)	35 (63.64%)	45 (75.00%)		
Brain metastasis						
Yes	36 (22.64%)	8 (18.18%)	10 (18.18%)	18 (30.00%)	2.98	0.23
No	124 (77.36%)	36 (81.82%)	45 (81.82%)	42 (70.00%)		

ECOG PS, Eastern Cooperative Oncology Group performance status.

### 2.2 Interventions

Group A: Platinum-based chemotherapy consisting of either carboplatin (AUC 5) or cisplatin (75 mg/m^2^) combined with pemetrexed (500 mg/m^2^) was administrated intravenously on day 1 of each 21-day cycle for 4–6 cycles, followed by maintenance pemetrexed treatment. Group B: anti-PD-1 monoclonal antibody (200mg) plus platinum-based chemotherapy were used on day one of each 21-day cycle for 4–6 cycles, followed by maintenance anti-PD-1 monoclonal antibody plus pemetrexed treatment. Among 55 patients in this group, 26 received camrelizumab, 8 tislelizumab, 13 sintilimab, and 8 pembrolizumab. Group C: bevacizumab 15 mg/kg plus platinum-based chemotherapy once every 3 weeks intravenously for four to six cycles, followed by maintenance bevacizumab plus pemetrexed treatment. Patients were required to complete ≥ 2 treatment cycles and undergo at least one efficacy assessment.

### 2.3 Study outcomes and endpoints

The effectiveness and safety data were collected. Tumor response was evaluated by investigators according to RECIST version 1.1 criteria. Evaluation indicators included complete response (CR), partial response (PR), stable disease (SD), and progressive disease (PD). The objective response rate (ORR) = (CR+PR)/total number of cases × 100% and the disease control rate (DCR) = (CR+PR+SD)/total number of cases × 100% were calculated. Progression-free survival (PFS) was defined as the time from the first-line treatment until confirmation of PD, death, or last follow-up. OS was defined as the time from the first-line treatment to death or the last follow-up. The National Cancer Institute CTCAE (version 5.0) was used to grade adverse events (AEs). Patients were consulted and followed up in the inpatient and outpatient system of the Henan Cancer Hospital until May 2023.

### 2.4 Statistical analysis

Chi-Square or Fisher’s exact probability analysis was used to identify the difference of the incidence of adverse reactions between groups. Kaplan-Meier analysis was used to estimate the OS or PFS, and log-rank test was used to identify differences between groups. The Cox regression model was fitted based on multiple risk factors and OS or PFS, and Hazard Ratios (HR) were estimated to identify the relationship between these risk factors and the outcomes. GraphPad Prism 8.0 software was used to plot survival curves. The significant level alpha was set to be 0.05. All statistical analysis was conducted using SPSS software v.26.0.

## 3 Results

### 3.1 Patient characteristics

159 patients were included in this analysis, including 44 patients in Group A (27.67%), 55 patients in Group B (34.59%), and 60 patients in Group C (37.74%). There was no substantial difference in baseline characteristics among the three groups of patients (P>0.05, **Table 1**). Of 159, 47 (29.56%) patients were female. The median age was 61 years (range 32-85), with 59 (37.11%) patients aged ≥ 65 years. 136 patients (85.53%) had ECOG PS of 0-1. All patients had stage IV LUAD at diagnosis with negative PD-L1 expression and without sensitizing EGFR, ALK, ROS1, Met, Ret or Braf V600E mutations, thereby ERBB2 (13, 8.18%), Kras (27, 16.98%) and TP53 (27, 16.98%) mutations were allowed. Most of the patients had liver (24, 15.09%), brain (36, 22.64%), and bone metastasis (56, 35.22%) (**Table 1**).

### 3.2 Clinical response

**Figure 2:**
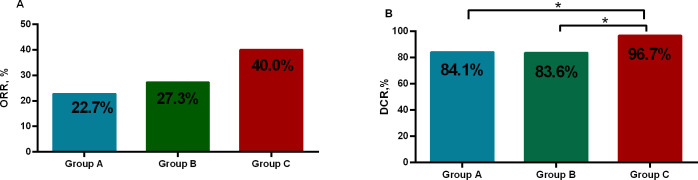
The objective response rate (ORR) and the disease control rate (DCR). **(A)** ORR of the three groups was similar (P>0.05). **(B)** DCR for patients in group C was significantly higher than group A, B (P<0.05). *p<0.05.

All the patients were included in the response assessment, with an ORR of 30.81% and a DCR of 88.68%. DCR for patients in group C (96.67%) was significantly higher than group A (84.09%) and B (83.64%) (P=0.04), but there was no difference between group A and B (P=0.95). ORR for patients in group C (40%) was higher than for patients including group A (22.73%) and B (27.27%), whereas the difference was not significant among them (P=0.13) (**Tables 2**, **3**, **Figure 2**).

**Table 2:** Summary of tumor response.

Group	A	B	C	χ²	P
CR(n)	0	0	0	–	–
PR(n)	10	15	24	–	–
SD(n)	27	31	34	–	–
PD(n)	7	9	2	–	–
ORR(%)	22.73	27.27	40	4.05	0.13
DCR(%)	84.09	83.64	96.67	6.13	0.04

CR, complete response; PR, partial response; SD, stable disease; PD, progressive disease; ORR, Objective response rate; DCR, Disease control rate.

**Table 3:** Multiple comparisons of disease control rate.

Groups	DCR	χ^2^	P
A vs B	37 (84.09%) vs 46 (83.64%)	0.004	0.95
A vs C	37 (84.09%) vs 58 (96.67%)	5.08	0.02
C vs B	58 (96.67%) vs 46 (83.64%)	5.63	0.02

### 3.3 Survival analysis

**Figure 3:**
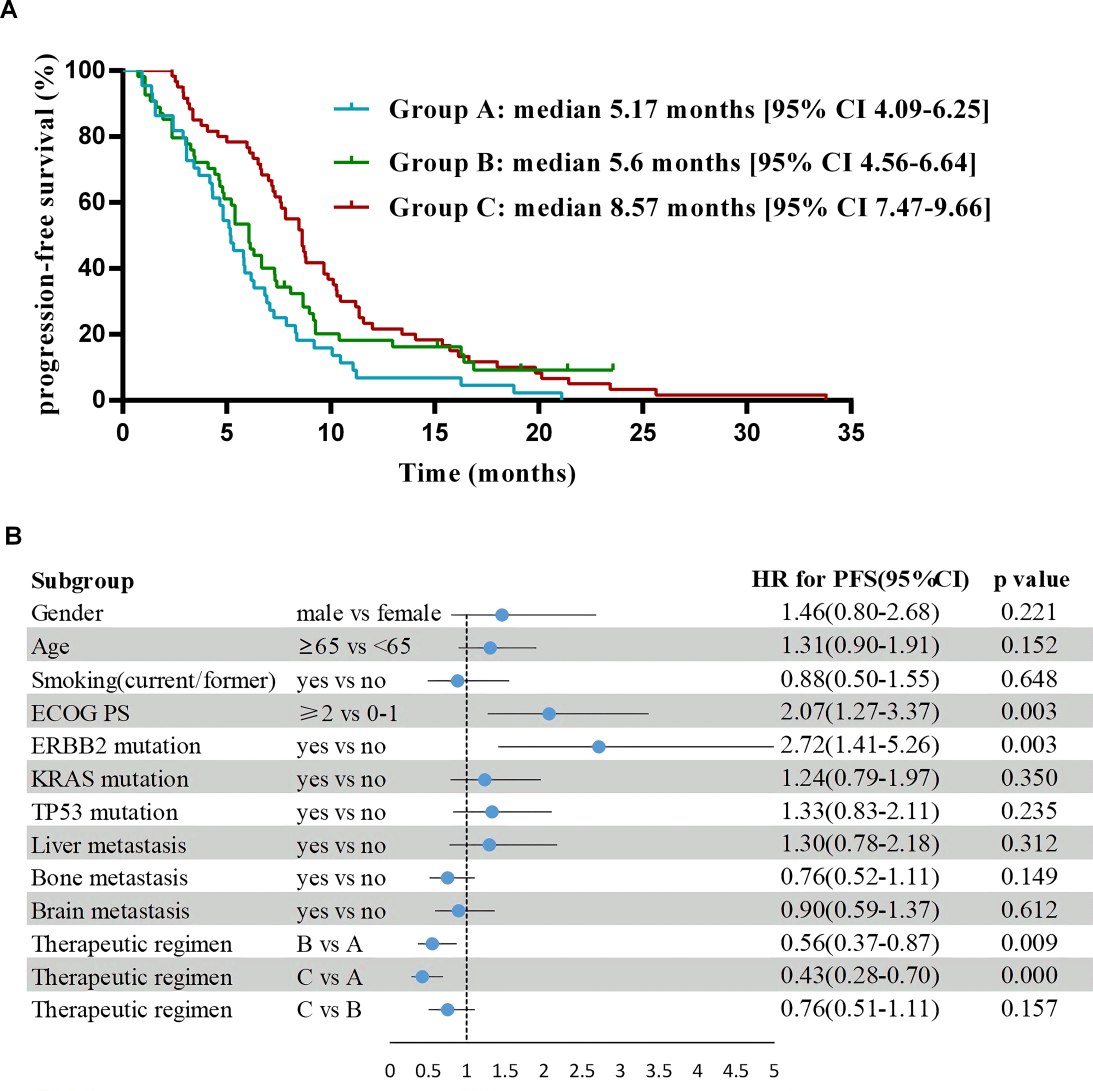
Progression-free survival and forest plot of hazard ratios for its risk factors. **(A)** Kaplan-Meier estimates of progression-free survival. **(B)** Logistic regression analyses to identify risk factors of progression-free survival in comparison to the control subjects after adjusting for gender, age, smoking (yes/no), ECOG PS, ERBB2 mutation, KRAS mutation, TP53 mutation, liver metastasis (yes/no), bone metastasis (yes/no), brain metastasis (yes/no) and therapeutic regimens.

**Figure 4:**
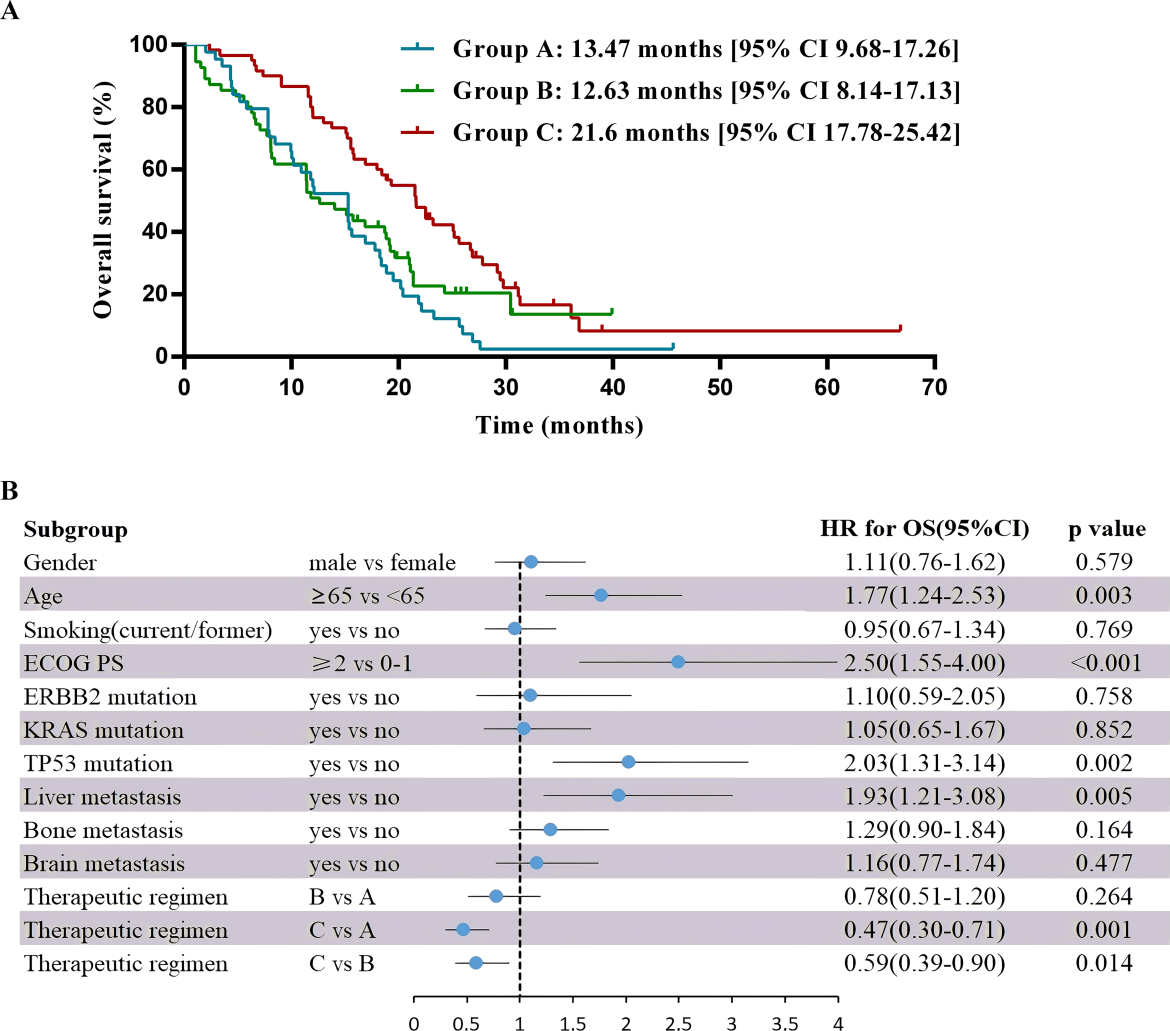
Overall survival and multivariable analysis of it. **(A)** Kaplan-Meier estimates of overall survival. **(B)** Forest plot of multivariable Cox proportional-hazard models for overall survival with hazard ratio for each of the factors included: gender, age, smoking, ECOG PS, ERBB2 mutation, KRAS mutation, TP53 mutation, liver metastasis, bone metastasis, brain metastasis and therapeutic regimens.

As of May 2023, the median follow-up time was 30.9 months. For the entire cohort, median PFS was 6.67 months [95% CI 5.83-7.51], and the median OS was 15.83 months [95% CI 13.46-18.21]. For PFS in pairwise comparison, group B and C were statistically superior to group A (B vs A median 5.6 months [95% CI 4.56-6.64] vs 5.17 months [95% CI 4.09-6.25]; hazard ratio (HR) 0.56 [95% CI 0.37-0.87], P = 0.009; C vs A median 8.57 months [95% CI 7.47-9.66] vs 5.17 months [95% CI 4.09-6.25]; HR 0.43 [95% CI 0.28-0.7], P< 0.001), whereas no significant difference was found between group B and C (HR 0.76 [95% CI 0.51-1.11], P= 0.16) (**Figure 3**). OS was significantly longer in group C versus the other ones (C vs B median 21.6 months [95% CI 17.78-25.42] vs 12.63 months [95% CI 8.14-17.13]; HR 0.59 [95% CI 0.39-0.9], P = 0.01; C vs A median 21.6 months [95% CI 17.78-25.42] vs 13.47 months [95% CI 9.68-17.26], HR 0.47 [95% CI 0.3-0.71], P= 0.001), but no substantial difference was noted between group A and B (HR 0.78 [95% CI 0.51-1.2], P= 0.26) (**Figure 4**).

### 3.4 Prognostic factors

Multivariate analysis showed that the ECOG PS score and treatment regimen were factors influencing PFS as well as OS. The prognostic factor analysis showed that P and HR for PFS favored patients receiving therapeutic regimen such as group B or C with ECOG PS 0–1 and negative ERBB2 (P<0.05), and P and HR for OS favored patients receiving therapeutic regimen such as group C with ECOG PS 0–1 and aged < 65 years but without liver metastasis and TP53 mutation (P<0.05) (**Figures 3**, **4**).

We evaluated the impact of TP53, KRAS and ERBB2 mutations on PFS and OS across different subgroups. The results revealed that patients with TP53 mutations other than KRAS and ERBB2 in group C exhibited significantly worse survival outcomes [HR for PFS: 2.06 (95%CI 1.03-4.09); HR for OS: 5.8 (95%CI 2.66-12.66)]. In contrast, the mutation status of TP53, ERBB2 and KRAS in group B did not show a significant association with PFS and OS (**Tables 4**, **5**).

**Table 4:** Biomarkers associated with progression-free survival.

Group			HR	95%CI	*P*
A	ERBB2 mutation	yes vs no	5.55	1.50-20.56	0.01
	KRAS mutation	yes vs no	1.03	0.43-2.46	0.95
	TP53 mutation	yes vs no	2.17	0.74-6.31	0.16
B	ERBB2 mutation	yes vs no	2.02	0.84-4.88	0.12
	KRAS mutation	yes vs no	1.30	0.61-2.76	0.50
	TP53 mutation	yes vs no	0.81	0.41-1.61	0.55
C	ERBB2 mutation	yes vs no	1.41	0.43-4.65	0.58
	KRAS mutation	yes vs no	1.61	0.84-3.08	0.15
	TP53 mutation	yes vs no	2.06	1.03-4.09	0.04

**Table 5:** Biomarkers associated with overall survival.

Group			HR	95%CI	*P*
A	ERBB2 mutation	yes vs no	2.86	0.83-9.86	0.10
	KRAS mutation	yes vs no	2.74	1.10-6.82	0.03
	TP53 mutation	yes vs no	4.02	1.32-12.27	0.01
B	ERBB2 mutation	yes vs no	0.95	0.34-2.69	0.93
	KRAS mutation	yes vs no	1.17	0.49-2.76	0.73
	TP53 mutation	yes vs no	1.28	0.62-2.66	0.51
C	ERBB2 mutation	yes vs no	1.13	0.40-3.18	0.81
	KRAS mutation	yes vs no	0.75	0.36-1.58	0.45
	TP53 mutation	yes vs no	5.80	2.66-12.66	0.00

### 3.5 Safety

All 159 patients completed at least two cycles of treatment. Treatment-emergent AEs occurred in all patients enrolled, but most (120 patients, 75.47%) occurrences were grade 1-2, and all the AEs were manageable without any fatal toxicities. 3 patients (5%) in group C were reported epistaxis of grade 1 or 2, which was often caused by bevacizumab.

Treatment-related AEs of grade 3–4 were reported in 39 patients (24.53%). The most common grade 3–4 AEs were anemia, decreased neutrophil, white blood cell or platelet counts, chemotherapy-induced nausea and vomiting (CINV) and hepatic dysfunction. The grade 3 or 4 immune-related AEs (irAEs) including rash or itching (2 patient, 3.64%), thyroid dysfunction (1 patient, 1.82%), adrenal insufficiency (1 patient, 1.82%), checkpoint inhibitor pneumonitis (1 patient, 1.82%) and colitis (1 patient, 1.82%) resulted from the ICI-mediated activation of the immune system in group B, but total treatment-emergent AEs of grade 3–4 occurred in a similar proportion of patients in each group (P>0.05) (**Table 6**).

**Table 6:** Occurrence of adverse reactions.

Adverse reaction event	≥ Level 3			P
	Group A	Group B	Group C	
Anemia	4 (9.09%)	4 (7.27%)	6 (10%)	0.93
Leukopenia	2 (4.54%)	1 (1.82%)	4 (6.67%)	0.45
Neutropenia	3 (6.81%)	0	5 (8.33%)	0.10
Thrombocytopenia	3 (6.81%)	1 (1.82%)	1 (1.67%)	0.26
Hepatic dysfunction	5 (11.36%)	1 (1.82%)	2 (3.33%)	0.73
CINV	4 (9.09%)	2 (3.64%)	2 (3.33%)	0.35
irAEs	0	6 (10.9%)	0	0.003
At least one treatment-related AE	16 (36.36%)	11 (20%)	12 (20%)	0.53

CINV, chemotherapy-induced nausea and vomiting; irAEs, immune-related adverse events.

## 4 Discussion

LUAD is a serious threat to human life. In particular, the prognosis for advanced patients is very poor, with the 5-year survival rate being exceedingly low (1, 2, 14). Clinical data shows that the combination of bevacizumab with chemotherapy versus chemotherapy alone can result in a better anti-tumor effect and delay drug resistance with controllable adverse reactions, thus such combination therapy is now a standard first-line treatment for advanced LUAD (4–6). In recent years, strategies using ICIs, which can enhance antitumor immune responses, have revolutionized the LUAD therapeutic landscape (15, 16), and the combination of anti-PD1 or anti-PD-L1 and platinum chemotherapy achieved better survival outcomes than chemotherapy alone in several randomized controlled trials (RCTs) (17–20), however, not all patients can benefit from immunotherapy, and PD-L1 expression is the most valuable biomarker for predicting the efficacy of immunotherapy (21). While multiple studies have demonstrated improved immunotherapy response in LUAD patients with elevated PD-L1 expression, a substantial proportion of PD-L1-negative cases represents a clinically significant population that warrants further investigation (9, 11, 15–20). The optimal treatment approach for patients with PD-L1-negative LUAD was not defined yet, and the real-world evidence published regarding these patients remained scarce, thus most of the data available came from subgroup or pooled analyses of different clinical trials (18–20). Promising results of adding pembrolizumab to chemotherapy has been reported from keynote-189 trial in all subgroups including the PD-L1-negative (8, 17), and the pooled analysis demonstrated a substantial clinical benefit (18), but the exploratory analyses in PD-L1-negative patients of Impower 150 trial revealed similar median OS in the three subgroups including atezolizumab and bevacizumab plus carboplatin-paclitaxel (ABCP), atezolizumab plus carboplatin-paclitaxel (ACP) and bevacizumab plus carboplatin-paclitaxel (BCP) (13). A meta-analysis of first-line immunotherapy combinations for advanced NSCLC demonstrated that the addition of bevacizumab to chemotherapy plus ICI does not provide significant survival benefits over chemotherapy plus ICI alone for PD-L1-negative patients, while also increasing toxicity and treatment complexity (22). Given the lack of evidence supporting the combination of chemotherapy, ICI, and bevacizumab as a recommended first-line treatment strategy, no participants received this regimen in our study. Tislelizumab and sintilimab also demonstrated the lack of clear benefit for first-line treatment of non-squamous NSCLC patients with PD-L1 negative and driver-gene wildtype (11, 23). Since most clinical trials of immunotherapy have not targeted PD-L1-negative patients with LUAD, only the results of subgroups analysis with limited statistical power are available and should be carefully considered. Therefore, the optimal therapy of LUAD patients with PD-L1-negative expression needs to be further explored in real-world studies. Approved first-line treatment strategies for LUAD patients with PD-L1 negative expression and without diver-gene mutations now include chemotherapy, immunochemotherapy and bevacizumab plus chemotherapy in the absence of head-to-head comparisons. To our knowledge, our study represents the first real-world analysis and the longest follow-up describing optimal first-line treatment options for PD-L1 negative LUAD patients with no known driver mutation.

Diver gene mutations including EGFR, ALK, ROS1, Met, Ret or Braf V600E are detected in approximately 50-70% of patients with LUAD (23–25), and standard first-line treatments for these patients were targeted therapies, thus these patients were not included in our study. Of the 1170 total LUAD patients randomly sampled from the database of the Hospital from January 2015 to May 2023, only 159 patients (13.59%) met the inclusion criteria and were successfully enrolled in this analysis, and the incidence was consistent with previous reports (23–25). In this retrospective cohort study, we conducted a head-to-head comparison of the clinical effectiveness and safety for these standard treatment regimens including chemotherapy alone, immunochemotherapy and bevacizumab plus chemotherapy. Here, the median PFS was 6.67 months [95% CI 5.83-7.51] and the median OS was 15.83 months, representing a poorer prognosis in real-world compared to RCTs (4–6, 8–11).

Bevacizumab plus chemotherapy demonstrated promising results in the present study. This regimen achieved much longer OS than ICI plus chemotherapy and chemotherapy alone, which could be considered the gold standard endpoint for identifying patients benefiting most from this regimen. Both bevacizumab and ICIs substantially extended the PFS of chemotherapy, thus the combination regimens could delay treatment resistance, which were consistent with previous evidence (4–6, 8–11). Compared to ICI plus chemotherapy, bevacizumab plus chemotherapy led to an improvement in PFS, but failed to show significant superiority. PFS was affected by multiple factors, including the patient population, imaging methods, and concomitant treatment, therefore it was only used as a surrogate marker of OS in some clinical trials and not always consistent with OS (26–28). The bevacizumab plus chemotherapy was also associated with a higher DCR than ICI plus chemotherapy and chemotherapy alone. In addition, we found that the ICI plus chemotherapy failed to show superior OS, DCR and ORR compared with chemotherapy alone in PD-L1 negative LUAD patients (HR 0.78 [95% CI 0.51-1.2], P= 0.264), and this findings were consistent with the subgroup analysis of tislelizumab and sintilimab, but contrary to subgroup and pooled analysis of pembrolizumab in the absence of head-to-head controlled comparisons. Given these results it is not clear that the differences in the structure and efficacy of ICIs and survival benefits between global populations and Chinese patients are clinically meaningful. Moreover, only 8 (14.55%) patients in this study accepted pembrolizumab plus chemotherapy, which might contribute to such differences. Therefore, we should focus on exploring the immunosuppressive microenvironment, and further research, particularly phase III prospective RCTs comparing treatment options in PD-L1-negative patients are urgently required.

Multivariate analysis showed that the ECOG PS score and treatment regimen were factors influencing PFS as well as OS. P and HR for PFS favored patients receiving therapeutic regimen such as group B or C with ECOG PS 0–1 and negative ERBB2 (P<0.05), and P and HR for OS favored patients receiving therapeutic regimen such as group C with ECOG PS 0–1 and aged < 65 years but without liver metastasis and TP53 mutation (P<0.05). Our findings indicated that bevacizumab plus chemotherapy brought more satisfactory efficacy and favorable survival to PD-L1-negative patients with treat-naïve advanced LUAD without common gene alterations than ICI plus chemotherapy and chemotherapy alone. Subgroup analysis revealed that TP53 mutations served as an independent adverse prognostic marker in bevacizumab-based chemotherapy group, aligning with previous studies (29, 30). However, TP53, ERBB2 and KRAS mutations showed no significant association with prognosis in the chemoimmunotherapy group. STK11 and KEAP1 mutations are also not reliable predictors of response to ICI therapy, given their association with a non-inflamed tumor microenvironment (TME) and frequent co-occurrence with mutations such as KRAS (31–33). Moreover, there is no evidence to date indicating that STK11 and KEAP1 mutations impact outcomes with chemotherapy or bevacizumab (31, 33). In this study, a technical limitation exists wherein earlier cohort patients (predominantly 2015-2018) were primarily tested with PCR-based methods rather than comprehensive NGS panels, preventing full assessment of non-core driver mutations such as TP53, KRAS, STK11 and KEAP1. Consequently, the observed TP53 and KRAS mutation rates were lower than these in contemporary NGS-based studies (29–33), and STK11/KEAP1 status could not be analyzed.

The safety profile for bevacizumab plus chemotherapy was previously reported (4–6) and no safety signals were observed in our study, supporting the well-established and manageable AE profile for bevacizumab. Grade 1 or 2 epistaxis occurred in 3 patients (5%) receiving bevacizumab plus chemotherapy in this study, prompting the recommendation to avoid its use in high-bleeding-risk populations.

## 5 Conclusion

In summary, our study represents the first real-world analysis and the longest follow-up describing optimal first-line treatment options for PD-L1-negative LUAD patients without common driver gene alterations. Our findings suggest that bevacizumab plus chemotherapy appears the most effective therapeutic strategy for this patient population in terms of OS and DCR, respectively. Further research, particular prospective phase III RCTs comparing treatment options in PD-L1-negative patients are required.

## Data Availability

The original contributions presented in the study are included in the article/supplementary material. Further inquiries can be directed to the corresponding authors.
